# Endoscopic Gliding Forehead Lift: A Brow Shaping Method to Lift the Medial and Lateral Brow

**DOI:** 10.1007/s00266-025-05027-z

**Published:** 2025-06-17

**Authors:** Apinut Wongkietkachorn, Nuttapone Wongkietkachorn

**Affiliations:** Plastic and Reconstructive Surgery Unit, QPrime Surgical Center, Bangkok, Thailand

**Keywords:** Eyebrows, Forehead, Endoscopes, Hemostatics, Lifting, Rhytidoplasty, Sutures, Retrospective studies

## Abstract

**Introduction:**

The gliding browlift has gained popularity as a technique to elevate the lateral brow. However, in many Asian patients, achieving the desired brow shape often necessitates lifting both the medial and lateral aspects. The endoscopic gliding forehead lift represents an evolution of the gliding browlift, tailored to address these specific anatomical and aesthetic considerations. This study aims to describe the surgical technique and evaluate objective outcomes of brow reshaping six months postoperatively.

**Methods:**

A retrospective study was performed on patients who underwent endoscopic gliding forehead lifts between July 2022 and June 2024. The surgical technique for the endoscopic gliding forehead lift is outlined in detail. Objective outcomes were assessed using measurements of the brow-to-pupil distance (BPD), brow-to-lateral canthus distance (BLCD) and brow-to-medial canthus distance (BMCD) preoperatively and at six months post-surgery.

**Results:**

A total of 100 patients (200 eyebrows) were included in the study. The BPD increased from 19.0 [17.0, 21.0] mm preoperatively to 24.0 [22.0, 26.0] mm at six months postoperatively (*p *< 0.001). The BLCD increased from 21.0 [20.0, 23.0] mm preoperatively to 28.0 [27.0, 29.0] mm at six months postoperatively (*p *< 0.001). The BMCD increased from 18.0 [16.0, 20.0] mm to 21.0 [19.0, 23.0] mm at six months postoperatively (*p *< 0.001). No cases of hematoma or frontal branch of facial nerve palsy were observed in this study.

**Conclusion:**

The endoscopic gliding forehead lift is an effective technique for shaping and lifting both the medial and lateral brows.

**Level of Evidence IV:**

This journal requires that authors assign a level of evidence to each article. For a full description of these Evidence-Based Medicine ratings, please refer to the Table of Contents or the online Instructions to Authors www.springer.com/00266.

**Supplementary Information:**

The online version contains supplementary material available at 10.1007/s00266-025-05027-z.

## Introduction

The gliding browlift [1, 2] is becoming an increasingly popular procedure worldwide. The glidelift [3] is also the further development of the gliding browlift, designed to shape the brow more effectively. However, the brows of Asian patients often require lifting both medially and laterally to achieve the desired aesthetic consistent with local concepts of beauty. In the central forehead, strong fibrous structures connect the dermis to the underlying layers along the crease area [4], posing challenges for safe dissection using previously described methods [1, 3]. The endoscopic gliding forehead lift further refines the gliding browlift technique, making it more suitable for Asian patients. This study aims to describe the technique and evaluate the objective outcomes of brow shaping six months postoperatively.

## Methods

### Study Design and Participants

A retrospective study was conducted on patients who underwent endoscopic gliding forehead lifts between July 2022 and December 2024. The inclusion criterion was undergoing an endoscopic gliding forehead lift. All patients were thoroughly informed about the use of a hemostatic net before the procedure. Exclusion criteria included prior browlifts, forehead lifts, thread lifts, or illegal filler injections.

### Endoscopic Gliding Forehead Lift Procedure

Patients were consulted in the upright position. The brow position and shape were adjusted manually using fingers to simulate the expected results. Once the patients agreed on the desired brow position and shape, the lifting vector and brow position were documented. The brow-to-pupil distance (BPD), brow-to-lateral canthus distance (BLCD) and brow-to-medial canthus distance (BMCD) were measured to assess the patient’s anatomy preoperatively. Any preexisting asymmetry in the brows was explained and emphasized to the patients.

The procedure was performed with the patient in the supine position under sedation. Video 1 demonstrates the initial steps of the procedure. An approximate 3 cm incision was marked along the anterior hairline of the forehead, and a 2.5 cm incision was marked at the sideburn. The incisions were infiltrated with local anesthesia, and a tumescent solution was applied to the entire forehead area. The skin was incised and initially elevated from the frontalis muscle. An endoscope was introduced, and dissection was performed from the midline across the entire forehead. The dissection was carefully inspected, ensuring the frontalis muscle was preserved.

Video 2 demonstrates the dissection in the temporal area. A 2.5 cm incision was made at the sideburn, and the endoscope was introduced to create a subcutaneous tunnel connecting to the forehead area. Then, a 5 mm opening was made in the superficial temporal fascia to access the deep layer just above the deep temporal fascia. Using Viterbo dissectors (straight and L-shaped, Faga Medical, Brazil), dissection was carried out in the deep layer, extending posteriorly toward the occipital area. Dissection beneath the superficial temporal fascia was performed to minimize the risk of hair loss in the hair-bearing region. Following dissection, two distinct planes were created, as illustrated in Video 2: the superior plane anterior to the hairline in the subcutaneous layer and the inferior plane posterior to the hairline in the deep layer.

**Fig. 1:**
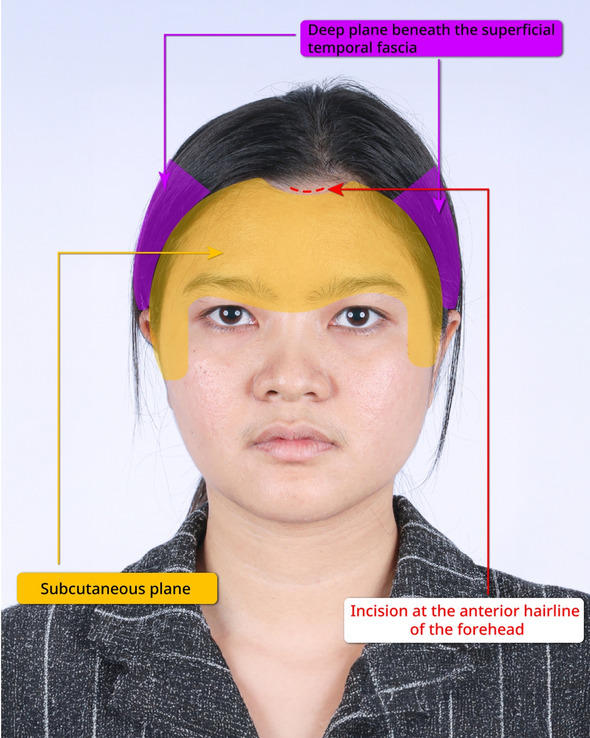
The illustration in frontal view of the dissection of the endoscopic gliding forehead lift procedure

**Fig. 2:**
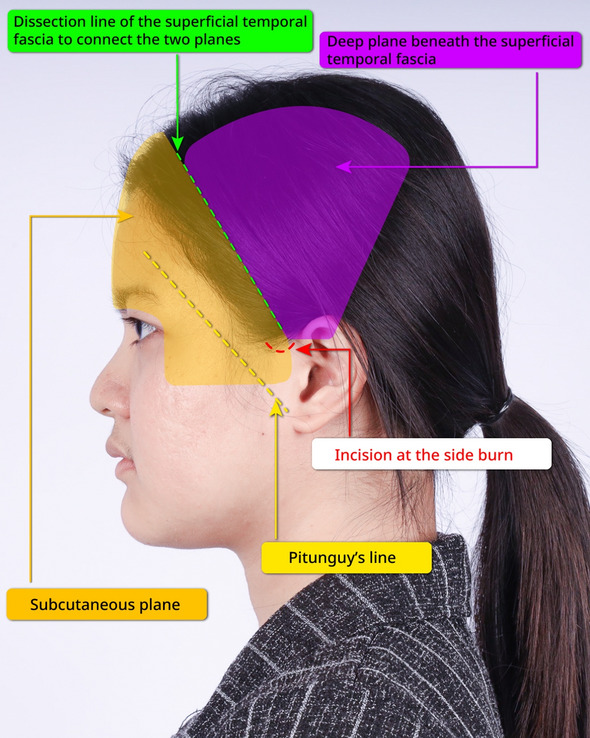
The illustration in lateral view of the dissection of the endoscopic gliding forehead lift procedure

Video 3 illustrates the connection of these two planes. A LigaSure device (Maryland Jaw, Medtronic, USA) was used to cut the superficial temporal fascia, enabling the connection. The endoscope provided visualization during this step. Once the planes were connected, the external skin was mobilized without bunching up anterior to the hairline, as the excess skin was redistributed into the hair-bearing region. This redistribution prevented the need for any incisions in the temporal area. Figures 1 and 2 summarize and illustrate the dissection of the endoscopic gliding forehead lift procedure.

Video 4 demonstrates the brow shaping and fixation technique. The brows were shaped and lifted to the patient’s desired position, starting with the lower side of the brow and then adjusting the higher side to achieve optimal symmetry. The brows were secured using 3-0 non-absorbable sutures for seven days, while the remaining area was stabilized with a hemostatic net using 5-0 non-absorbable sutures for two days.

### Post-operative Protocol

The surgical sites were covered with gauze and bandages for two days. The hemostatic net was removed on the third day, after which patients were permitted to wash their face and apply cream or lotion. Sutures were removed at one week. Patients were advised to avoid sunlight exposure for two months post-surgery. They were also informed that markings from the hemostatic net typically fade within 1–2 months without requiring additional medication, as previously reported by the authors [5].

### Outcomes

The primary outcome was the brow-to-pupil distance (BPD). Secondary outcomes included the brow-to-lateral canthus distance (BLCD), brow-to-medial canthus distance (BMCD), the rate of hematoma and the rate of frontal branch of facial nerve palsy. All distances were measured directly on the patients using a ruler. The BPD, BLCD, BMCD were measured preoperatively and at six months postoperatively. Because each brow required individualized lifting and shaping, analyses were conducted on each brow separately.

### Statistical Analysis

Data were analyzed using STATA/SE version 10.1. The Kolmogorov-Smirnov test was employed to assess data normality. Non-parametric data were reported as medians with interquartile ranges for continuous variables and as numbers with percentages for discrete variables. Differences in BPD, BLCD and BMCD were evaluated using the Wilcoxon signed-ranks test. All statistical tests were two-sided, with a *p*-value of less than 0.05 considered statistically significant.

## Results

A total of 100 patients (200 eyebrows) participated in the study. The mean age of the participants was 54.2 ± 10.1 years, and their mean body mass index (BMI) was 21.8 ± 6.6 kg/m^2^. Ninety percent of the patients were female. The prevalence of underlying conditions included 4.0% with diabetes mellitus, 6.0% with hypertension, and 5.0% with dyslipidemia. The brow-to-pupil distance (BPD) increased from 19.0 [17.0, 21.0] mm preoperatively to 24.0 [22.0, 26.0] mm at six months postoperatively (*p *< 0.001). The brow-to-lateral canthus distance (BLCD) increased from 21.0 [20.0, 23.0] mm preoperatively to 28.0 [27.0, 29.0] mm at six months postoperatively (*p *< 0.001). The brow-to-medial canthus distance (BMCD) increased from 18.0 [16.0, 20.0] mm to 21.0 [19.0, 23.0] mm at six months postoperatively (*p *< 0.001). No cases of hematoma or frontal branch of facial nerve palsy were observed in this study.

**Fig. 3:**
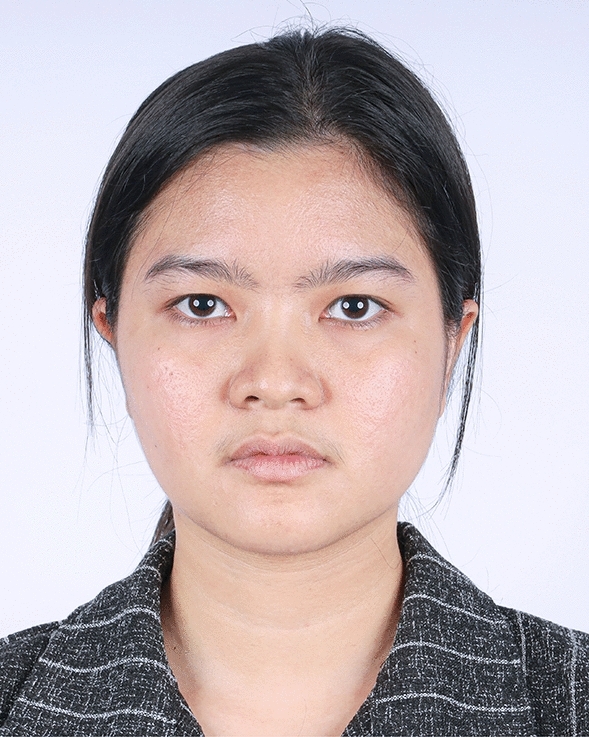
A 30-year-old female undergoing an endoscopic gliding forehead lift. She presented with a low brow-to-pupil distance, which contributed to a masculine appearance. She wanted to lift her brows to a higher position without changing the shape of her brows

**Fig. 4:**
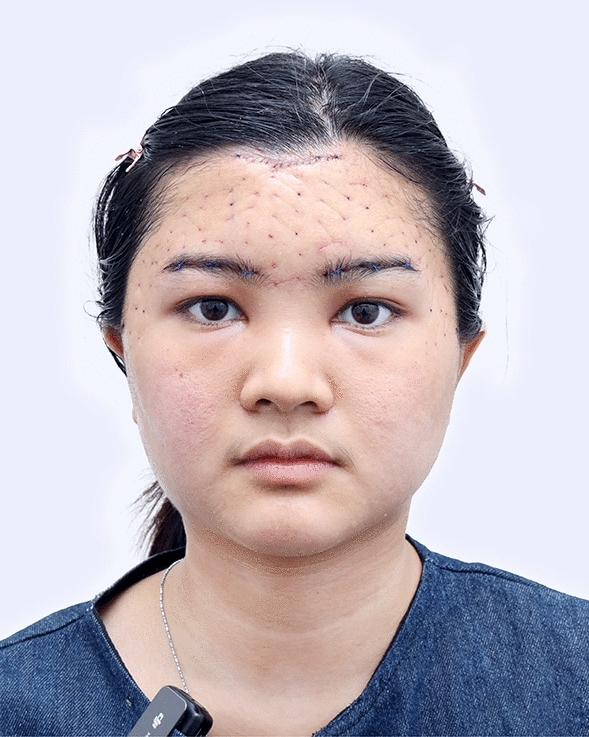
Three days after an endoscopic gliding forehead lift. The hemostatic net has been removed

**Fig. 5:**
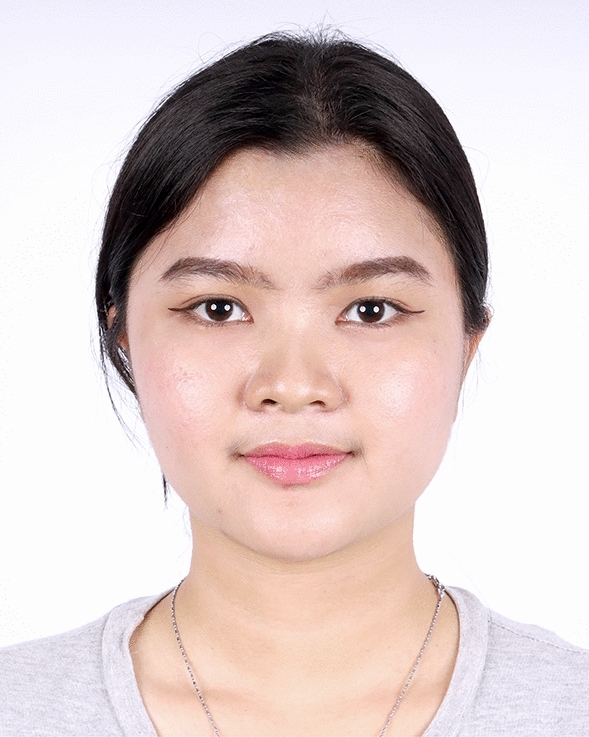
One year after an endoscopic gliding forehead lift in a 30-year-old female. The elevated brow highlights her double eyelid fold, creating a more feminine appearance

**Fig. 6:**
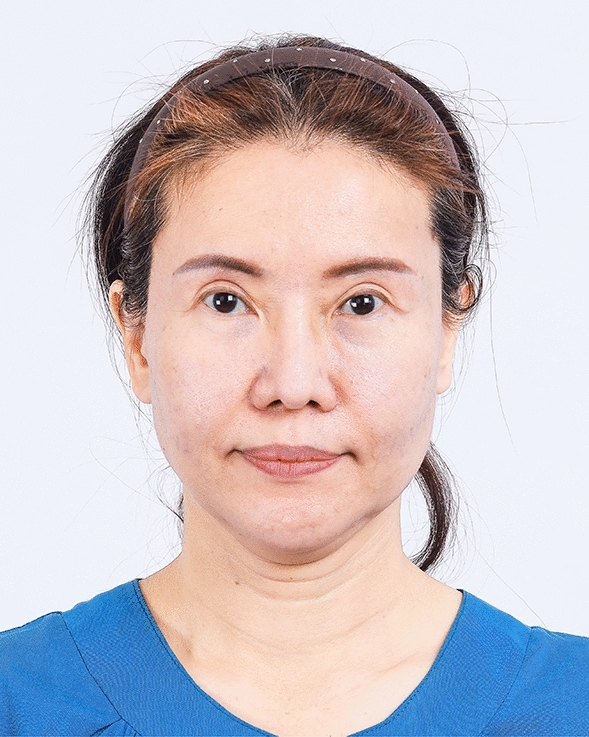
A 48-year-old female undergoing an endoscopic gliding forehead lift combined with a facelift. She wanted to lift both the medial and lateral parts of the brows but desired the lifting in the lateral part to be greater than the medial part

**Fig. 7:**
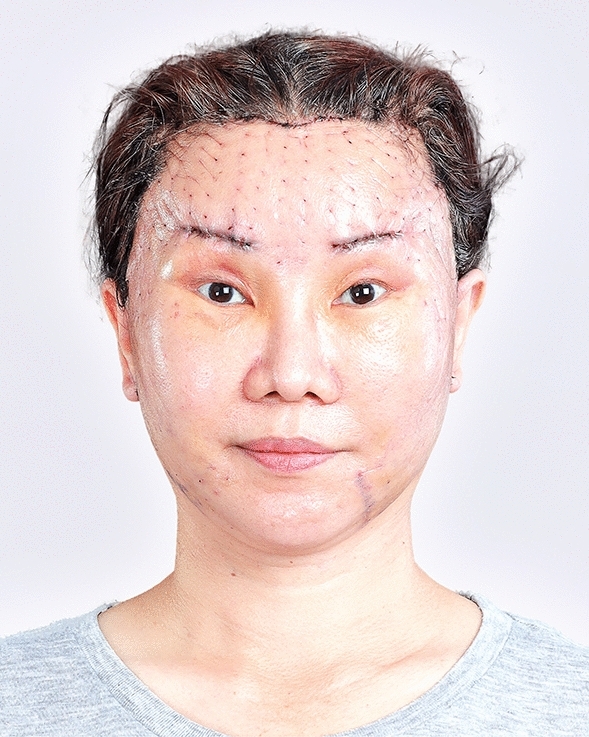
Three days after an endoscopic gliding forehead lift combined with a facelift. The hemostatic net has been removed

**Fig. 8:**
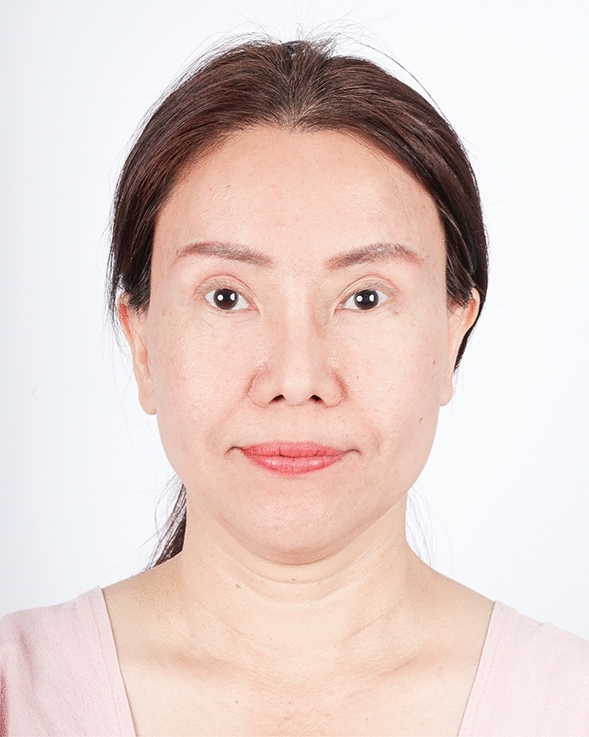
Two years after an endoscopic gliding forehead lift combined with a facelift. The brows were lifted laterally greater than medially

**Fig. 9:**
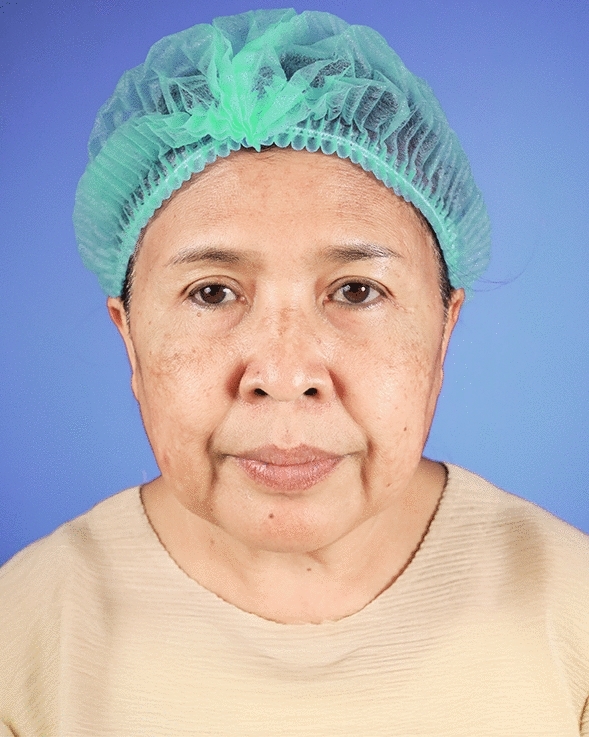
A 59-year-old female undergoing an endoscopic gliding forehead lift combined with a facelift. She came with ptotic brows that were horizontally straight. She wanted to lift the brow and change the shape from horizontally straight to more oblique and curved

**Fig. 10:**
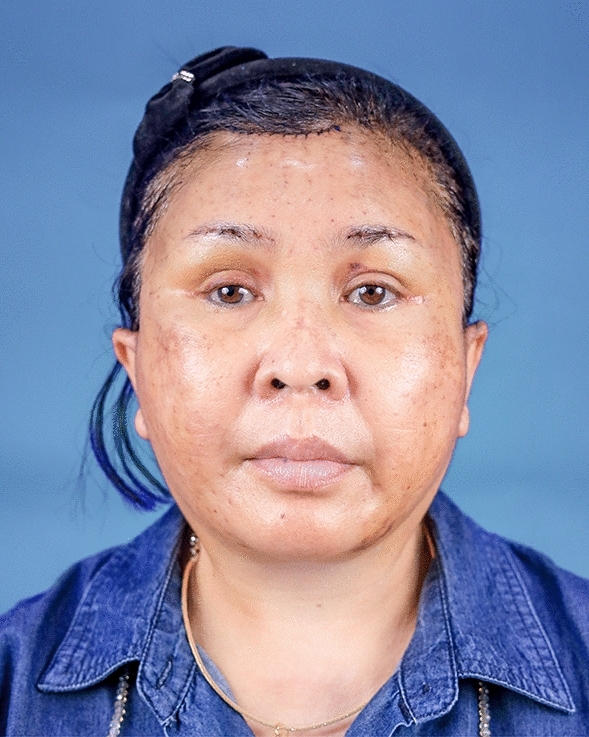
One week after an endoscopic gliding forehead lift combined with a facelift

**Fig. 11:**
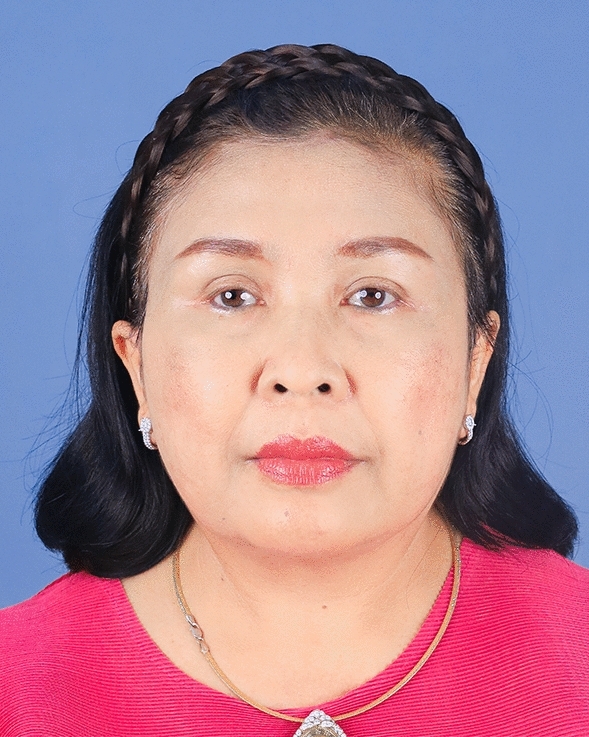
One year after an endoscopic gliding forehead lift combined with a facelift. The brows were lifted and the shape of the brow was changed from horizontally straight to more oblique and curved

**Fig. 12:**
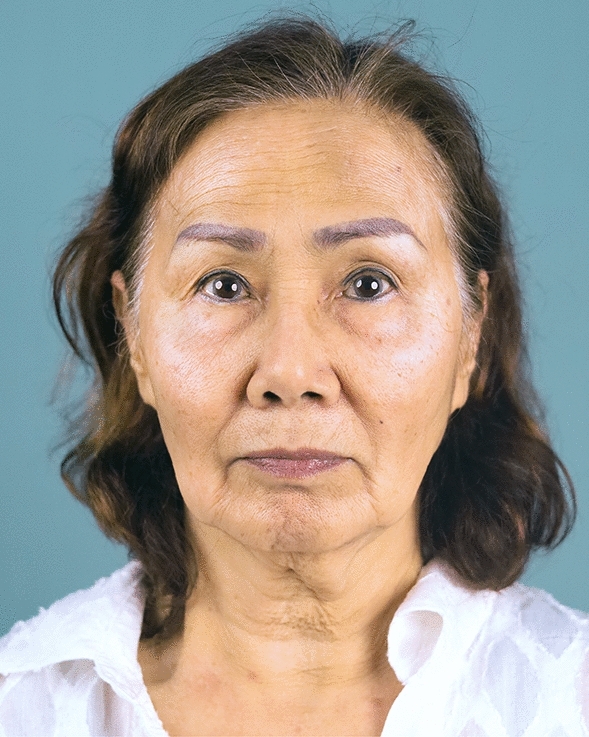
A 68-year-old female undergoing an endoscopic gliding forehead lift combined with a facelift and fat grafting. Her brows were asymmetrical, ptotic, and steeply angled shape

**Fig. 13:**
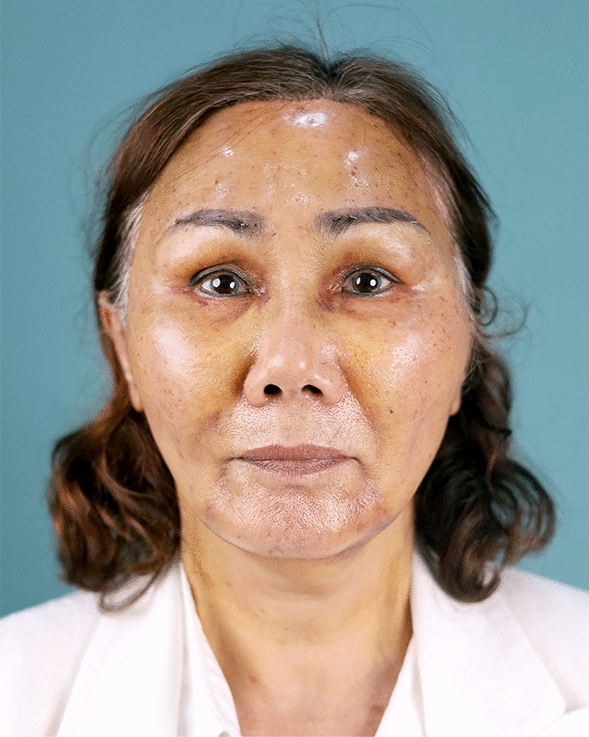
One week after an endoscopic gliding forehead lift combined with a facelift and fat grafting

**Fig. 14:**
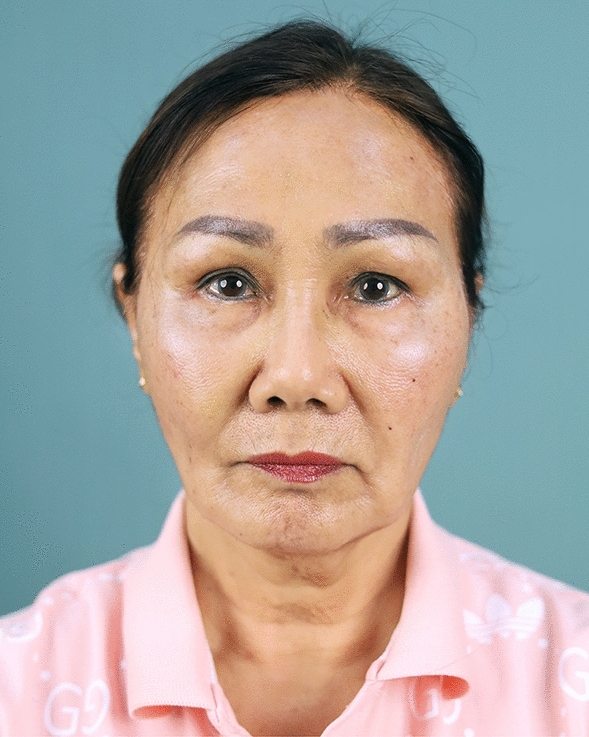
One year after an endoscopic gliding forehead lift combined with a facelift and fat grafting. The brows were elevated and appear more symmetrical. The shape of the brow was changed from steeply angled to curved

The examples of the outcomes achieved with the endoscopic gliding forehead lift in four different scenarios are illustrated in Figs. 3, 4, 5, 6, 7, 8, 9, 10, 11, 12, 13, 14. The first case shows both medial and lateral browlift without the change in the shape of the brows, as shown in Figs. 3, 4, 5. The second case shows both medial and lateral browlift, but the patient desired the lifting in the lateral part greater than the medial part as shown in Figs. 6, 7, 8. The third case shows both medial and lateral browlift with a significant change in the shape of the brow from horizontally straight to more oblique and curved, as shown in Figs. 9, 10, 11. The fourth case shows the correction of the asymmetrical brow with steeply angled shape to be the more symmetrical brows with curved shape as shown in Figs. 12, 13, 14.

## Discussion

The endoscopic gliding forehead lift is unique in its ability not only to “lift” the brow but also to “shape” it both medially and laterally. Subcutaneous dissection facilitates the mobilization of the brow and skin into the desired position with ease [6]. The hemostatic net provides multiple fixation points, allowing precise setting of the brow in the desired shape and location [1]. This distinct feature positions the method as a valuable addition to the repertoire of browlift techniques.

Achieving an appropriately shaped brow is critical. Lifting only the lateral portion of the brow may be unsuitable for some patients, as it can create an overly upslanted appearance. For Asian patients, who often have naturally upslanted eyes, lifting the brow solely in the lateral region may result in a pronounced “angry” expression [7]. Conversely, some browlift techniques lift the brow predominantly in the medial region over time, producing a “surprised” look [8]. The endoscopic gliding forehead lift addresses these issues by effectively shaping and lifting the brow both medially and laterally.

The endoscopic method ensures the preservation of the frontalis muscle. Strong fibrous structures connecting the dermis to the underlying layers in the frontal crease area [4] make dissection challenging when using the blunt technique described in the original gliding browlift [1]. Surgeons unfamiliar with the gliding browlift and its blunt instrumentation risk damaging the frontalis, potentially leading to abnormal brow movement. The use of an endoscope for clear visualization, combined with sharp dissection in the central portion of the frontalis, is essential for advancing the gliding browlift technique and achieving optimal outcomes.

The endoscopic gliding forehead lift results in smaller wounds compared to the traditional subcutaneous browlift [6]. This minimally invasive approach is a significant advancement, particularly for patients concerned about lengthy scars. First, the incision at the central hairline is approximately 3 cm long, significantly shorter than the full-length incision required when employing the traditional method [6]. Additionally, no incision is made in the temporal region, except for a discreet 2.5 cm incision at the sideburn, which contrasts with the longer anterior hairline incisions required for a temporal lift. Second, the sideburn incision is typically well-concealed by the patient’s hair, which naturally falls to cover the area. The sideburn incision also allows the dissection of the superior temporal fascia described above easily by approaching from downward. Third, the central incision serves multiple purposes: it allows for the removal of excess skin, improves forehead rhytides, reduces glabellar lines, lifts the medial brow, and prevents hairline elevation that could otherwise contribute to an aged appearance [6].

### Limitations

The use of an endoscope is required for achieving smaller wounds during the procedure. However, if an endoscope is not utilized, the procedure can be performed through larger incisions, using a retractor for traction and performing sharp dissection under direct visualization.

The goal of the endoscopic gliding forehead lift in this study was to position the brow in the patient’s desired shape and location, customized to suit each individual brow. The objective was not to achieve the maximum possible lift of the brow. Consequently, the differences in distances measured in this study cannot be interpreted as the maximum lifting capacity of the gliding browlift technique. In the authors’ opinion, the true success of a browlift lies in enhancing facial aesthetics by precisely positioning the brow, rather than achieving the greatest possible lift. Thus, the measured outcomes in this study primarily demonstrate the difference in brow position before and after the operation, as well as the stability of the results over time.

In practice, it is unnecessary to rely on a single method to lift the brow for every patient. The endoscopic gliding forehead lift is specifically designed to address both the medial and lateral brow, avoid elevating the central hairline, and prevent the creation of wounds in the temporal area. Careful case selection to determine the most suitable technique for each individual patient is strongly recommended.

## Conclusion

The endoscopic gliding forehead lift is an effective technique for shaping and lifting both the medial and lateral brows.

## Supplementary Information

Below is the link to the electronic supplementary material.

Supplementary file1. Video 1 The procedure's initial steps and the forehead dissection are demonstrated (MP4 94065 KB)

Supplementary file2. Video 2 The dissection in the temporal area and the creation of the two distinct planes are shown (MP4 91744 KB)

Supplementary file3. Video 3 The connection of the two distinct planes with a Ligasure device is shown (MP4 93953 KB)

Supplementary file4. Video 4 The brow shaping and fixation technique is shown (MP4 95426 KB)
